# Five Year Nationwide Incidence of Rhegmatogenous Retinal Detachment Requiring Surgery in Korea

**DOI:** 10.1371/journal.pone.0080174

**Published:** 2013-11-13

**Authors:** Sang Jun Park, Nam-Kyoung Choi, Kyu Hyung Park, Se Joon Woo

**Affiliations:** 1 Department of Ophthalmology, Seoul National University College of Medicine, Seoul National University Bundang Hospital, Seongnam, Republic of Korea; 2 Medical Research Collaborating Center, Seoul National University Hospital and Seoul National University College of Medicine, Seoul, Republic of Korea; 3 Institute of Environmental Medicine, Seoul National University Medical Research Center, Seoul, Republic of Korea; Massachusetts Eye & Ear Infirmary, Harvard Medical School, United States of America

## Abstract

**Purpose:**

To define the incidence and demographic characteristics of rhegmatogenous retinal detachment (RRD) requiring surgery in Korea.

**Design:**

Nationwide population-based retrospective study.

**Methods:**

Patients who underwent surgery for RRD from 2007 to 2011 were retrospectively identified using the diagnostic code for RRD and the surgical codes for retinal detachment surgeries in the national claim database. The average incidence rate of RRD during the 5-year period was estimated using the population data of the 2010 Census in Korea.

**Results:**

A total of 24,928 surgically treated RRD cases were identified. The average incidence of surgery requiring RRD was 10.39 cases per 100,000 person-years [95% confidence interval (CI), 10.26–10.52). The incidence in men (11.32 cases per 100,000 person-years; 95% CI: 11.13–11.51) was significantly higher than that in women (9.47 cases per 100,000 person-years; 95% CI: 9.29–9.64) (p<0.001). The incidence of surgery requiring RRD showed a bimodal distribution across age groups, with one peak (28.55 cases per 100,000 person-years; 95% CI: 27.46–29.67) representing patients between 65 and 69 years of age and the second peak (approximately 8.5 per 100,000 person-years) representing patients between 20 and 29 years of age. The male-to-female ratio was approximately 1.0 for the peak-incidence age groups, whereas the ratio was higher for the other age groups.

**Conclusions:**

The incidence of RRD in the Korean population was similar to that reported previously, with the peak incidence being lower than that in the Caucasian population. The age-specific RRD incidence pattern in Korea followed a bimodal distribution.

## Introduction

Rhegmatogenous retinal detachment (RRD) is a potentially blinding disease characterized by the separation of the inner neurosensory retina and the outer retinal pigment epithelium due to a break in the retina. RRD may require therapeutic surgery to prevent permanent loss of vision. Previous studies have reported an annual RRD incidence rate of 6.9–18.2 per 100,000 persons, which showed high variance across time and place.[1]–[4] Most of these studies estimated the RRD incidence in a specific regional population and not the nationwide population.[2], [5], [7]–[10], [14] Van de Put et al[13] provided a reliable and informative report of the RRD incidence in the nationwide population of the Netherlands. However, the study was limited in that the RRD incidence was estimated from retrospectively reviewed hospital records for a relatively short period (1 year). Only studies conducted in Scotland[12], [15] and Singapore[6] showed nationwide RRD incidence based on the national health claim database with relatively long study periods. The study conducted in Scotland[12], [15] reported the incidence of RRD in the Caucasian population during a recent 10-year period, and the study conducted in Singapore[6] reported the incidence of RRD in the Southeast Asian population in the mid-90s. To date, however, no nationwide study has reported the incidence of RRD in Northeast Asia, one of the most populous regions in the world, using an administrative database. Korea is situated in Northeast Asia and is a promising place for the estimation of the RRD incidence because the entire Korean population is registered under the National Health Insurance system, and it is possible to obtain reliable and accurate data regarding surgical RRD cases. Therefore, we conducted this nationwide study to evaluate the incidence of RRD cases between 2007 and 2011, which required surgical treatment in Korea, using the national health claim database.

## Methods

For this study, we used data recorded between 2007 and 2011 in the national health claim database obtained from the Health Insurance Review and Assessment service (HIRA) of Korea. The HIRA provided the data after de-identification before we could access the database. The study was approved by the Seoul National Bundang Hospital institutional review board and adhered to the tenets of the Declaration of Helsinki.

The Korean National Health Insurance (NHI) scheme covers approximately 97% of the Korean population and is a compulsory social insurance. Patients insured by the NHI pay approximately 30% of their total medical expenses, and hospitals are required to submit claims for the remaining 70% of the expenses related to inpatient and outpatient care. Claims are accompanied by data regarding diagnoses, procedures, prescription records, demographic information, and direct medical costs. The medical expenses of the Korean population not insured by the NHI are covered by the Medical Assistance Program (MAP) or the Medical Care for Patriots and Veterans Affairs Scheme. Claims from these 2 schemes are also reviewed by the HIRA. Therefore, the HIRA database is very extensive, containing information for the entire Korean population, personal information of patients, and medical records related to all medical claims made in Korea.[16], [17] Furthermore, Korean citizens tend to seek medical attention related to retinal disorders in Korea, because the country is known to have well-trained retinal specialists and several hospitals have well-equipped facilities for the treatment of retinal diseases. As Korean health care providers charge only ≤30% of the total medical costs of RRD-related medical care, Koreans do not seek RRD-related medical attention in other countries, as this would involve high costs and might be inconvenient.

All Korean residents receive a unique identification number (Korean Resident Registration Number, KRRN) at birth, which enables easy identification of every citizen. This is widely used in government programs and in the health care system and the HIRA database. Therefore, this database can be used to obtain the health care records and the demographic characteristics of RVO patients without any duplications or omissions.

Public access to the HIRA database is not allowed. The restricted access to the HIRA database might be allowed after approbation of the Deliberative Committee of HIRA when the requested study is in conformity with the common good. The Deliberative Committee of HIRA approved the present study to conditional use of the HIRA database from 2007 to 2011 as following statement. The HIRA database manages claims using the Korean Classification of Disease, sixth edition (KCD-6). The KCD-6 is similar to the International Classification of Disease (ICD-10), because KCD-6 is a modified version of the ICD-10 for the Korean health care system. The compulsory healthcare system in Korea covers RRD-related health care costs, including RRD surgeries. For this study, we included the surgery-requiring RRD cases and excluded RRD cases that did not require surgical treatment and other types of retinal detachment (RD) cases such as exudative RD, traction RD, RD with retinopathy of prematurity, RD after endophthalmitis, and RD after perforating and/or penetrating wound. Only those RRD cases in which both the diagnostic and surgical codes were simultaneously claimed were included in this study. The diagnostic code for RRD is H33.0 (RD with retinal break; H33.0 includes H33.00, H33.01, H33.02, H33.04, and H33.09). The surgical codes for RRD are S5130 (RD surgery), S5121 (vitrectomy, total), and S5122 (vitrectomy, partial). We excluded cases with the following diagnostic codes: H33.4 (traction detachment of retina), H35.01 (exudative retinopathy, Coats'disease), H35.1 (retinopathy of prematurity), H35.2 (other proliferative retinopathy, proliferative vitero-retinopathy), H44.0 (purulent endophthalmitis), H44.1 (other endophthalmitis: parasitis endophthalmitis not otherwise specified, sympathetic uveitis), H44.6 (retained intraocular foreign body, magnetic), H44.7 (retained intraocular foreign body, nonmagnetic), H45.1 (endophthalmitis in disease classified elsewhere), Q11.2 (microphthalmos), Q12 (congenital lens malformations), Q14 (congenital malformations of the posterior segment of the eye), S05.2 (ocular laceration and rupture with prolapse or loss of intraocular tissue), S05.3 (ocular laceration without prolapse or loss of intraocular tissue), S05.4 (penetrating wound of orbit with or without foreign body), S05.5 (penetrating wound of eyeball with foreign body), S05.6 (penetrating wound of eyeball without foreign body), and S05.7 (avulsion of eye). For those cases that had 2 or more claims with diagnostic codes for surgery-requiring RRD cases during the study period, the first claim in the database was defined as the incident time, and the patient was then counted as an incident case in that year.

The population at risk was defined as the entire population of Korea based on the Population and Housing Census (PHC) of 2010 available from the Korean Statistical Information Service (http://kosis.kr). The Korean Statistical Information Service of the Korean central government conducts the PHC every 5 years to obtain information regarding the size, distribution, and structure of population and housing in Korea. The PHC was conducted in 2005 and 2010, and the next PHC is scheduled for 2015. The Korean population was estimated at 47,990,761 individuals. Detailed demographics of the population are listed in Table 1.

**Table 1:** Demographics of Korea and Incidence of Rhegmatogenous Retinal Detachment in Korean Population from 2007 to 2011.

	Number of Korean Population^a^			Total		Men		Women		
Age group (years)	Total	Male	Female	No	Incidence^b^ (95% CI)	No	Incidence (95% CI)	No	Incidence (95% CI)	Male to Female Ratio
0–4	2219084	1142220	1076864	1	0.01 (0–0.13)	0	0.00 (0–0)	1	0.02 (0–0.05)	0.00
5–9	2394663	1243294	1151369	57	0.48 (0.35–0.60)	41	0.66 (0.46–0.86)	16	0.28 (0.14–0.41)	2.37
10–14	3173226	1654964	1518262	380	2.40 (2.15–2.64)	303	3.66 (3.25–4.07)	77	1.01 (0.79–1.24)	3.61
15–19	3438414	1826179	1612235	1097	6.38 (6.00–6.76)	794	8.70 (8.09–9.30)	303	3.76 (3.34–4.18)	2.31
20–24	3055420	1625371	1430049	1309	8.57 (8.10–9.03)	722	8.88 (8.24–9.53)	587	8.21 (7.55–8.87)	1.08
25–29	3538949	1802805	1736144	1491	8.43 (8.00–8.85)	730	8.10 (7.51–8.69)	761	8.77 (8.14–9.39)	0.92
30–34	3695348	1866397	1828951	1244	6.73 (6.36–7.11)	667	7.15 (6.61–7.69)	577	6.31 (5.79–6.82)	1.13
35–39	4099147	2060233	2038914	1316	6.42 (6.07–6.77)	783	7.60 (7.07–8.13)	533	5.23 (4.78–5.67)	1.45
40–44	4131423	2071431	2059992	1574	7.62 (7.24–8.00)	981	9.47 (8.88–10.06)	593	5.76 (5.29–6.22)	1.65
45–49	4073358	2044641	2028717	2150	10.56 (10.11–11.00)	1344	13.15 (12.44–13.85)	806	7.95 (7.40–8.49)	1.65
50–54	3798131	1887973	1910158	2960	15.59 (15.03–16.15)	1774	18.79 (17.92–19.67)	1186	12.42 (11.71–13.12)	1.51
55–59	2766695	1360747	1405948	3002	21.70 (20.92–22.48)	1552	22.81 (21.68–23.95)	1450	20.63 (19.56–21.69)	1.11
60–64	2182236	1057035	1125201	2999	27.49 (26.50–28.47)	1478	27.97 (26.54–29.39)	1521	27.04 (25.68–28.39)	1.03
65–69	1812168	833242	978926	2587	28.55 (27.45–29.65)	1194	28.66 (27.03–30.28)	1393	28.46 (26.97–29.95)	1.01
70–74	1566014	672894	893120	1653	21.11 (20.09–22.13)	719	21.37 (19.81–22.93)	934	20.92 (19.57–22.26)	1.02
75–79	1084367	410726	673641	763	14.07 (13.07–15.07)	277	13.49 (11.90–15.08)	486	14.43 (13.15–15.71)	0.93
80–84	595509	186008	409501	275	9.24 (8.14–10.33)	107	11.50 (9.32–13.68)	168	8.21 (6.96–9.45)	1.40
85–89	271166	74118	197048	61	4.50 (3.37–5.63)	25	6.75 (4.10–9.39)	36	3.65 (2.46–4.85)	1.85
90–94	78329	17770	60559	7	1.79 (0.46–3.11)	5	5.63 (0.69–10.56)	2	0.66 (0–1.58)	8.52
95–	17114	2848	14266	2	2.34 (0–6.25)	0	0.00 (0–0)	2	2.80 (0–7.52)	0.00
Total	47990761	23840896	24149865	24928	10.39 (10.26–10.52)	13496	11.32 (11.13–11.51)	11432	9.47 (9.29–9.64)	1.20

a Population of Korea was based on the 2010 census from the Korean Statistical Information Service.

b A unit for Incidence rate is per 100,000 person-years

The person-time incidence rates for 2007–2011 were calculated as the number of people who developed RRD divided by the total person-time at risk during the study period. Therefore, in this analysis, person-years were counted after the incident time. The annual RRD incidence rates were calculated as persons who developed RRD divided by the total population, based on the 2010 census. The age and sex-specific RRD incidence rates were estimated. A 95% confidence interval (CI) of the incidence rate was estimated based on the Poisson distribution. Male-to-female ratio for the RRD incidence rate was also estimated. All analyses were conducted using SAS, version 9.3 (SAS Institute, Inc, Cary, North Carolina).

## Results

A total of 24928 cases (50.3% women) of RRD were identified during the 5-year study period. The number of surgically treated RRD cases was 4833 (19.4%) in 2007, 4965 (19.9%) in 2008, 4895 (19.6%) in 2009, 5283 (21.2%) in 2010, and 4952 (19.9%) in 2011. The median age of the patients was 53 years (range, 1–96 years). The median ages of male and female cases were 51 years (range, 5–92 years) and 55 years (range, 1–96 years), respectively.

During the 5-year study period, the surgery requiring RRD incidence rate was 10.39 per 100,000 person-years (95% CI, 10.26–10.52). The 5-year surgery requiring RRD incidence rate in men and women was 11.32 per 100,000 person-years (95% CI, 11.13–11.51) and 9.47 per 100,000 person-years (95% CI, 9.29–9.64), respectively. The incidence of surgery requiring RRD in men was 1.20 times higher than that in women (p value<0.001) (Table 1).

The annual surgery requiring RRD incidence rates per 100,000 persons were 10.07 (95% CI, 9.79–10.35) in 2007, 10.35 (95% CI, 10.06–10.63) in 2008, 10.20 (95% CI, 9.91–10.49) in 2009, 11.01 (95% CI, 10.71–11.31) in 2010, and 10.32 (95% CI, 10.03–10.61) in 2011 (Table 2).

**Table 2:** Annual incidence (per 100,000 persons) of rhegmatogenous retinal detachment in Korean Population between 2007 and 2011.

	2007			2008			2009			2010			2011		
	Total	Men	Women	Total	Men	Women	Total	Men	Women	Total	Men	Women	Total	Men	Women
0–4	0.00	0.00	0.00	0.05	0.00	0.09	0.00	0.00	0.00	0.00	0.00	0.00	0.00	0.00	0.00
5–9	0.63	0.88	0.35	0.46	0.56	0.35	0.33	0.48	0.17	0.50	0.80	0.17	0.46	0.56	0.35
10–14	2.49	3.75	1.12	2.87	4.47	1.12	2.84	4.17	1.38	2.08	3.32	0.72	1.70	2.60	0.72
15–19	5.58	7.50	3.41	5.53	7.28	3.54	5.96	8.27	3.35	8.17	11.28	4.65	6.66	9.14	3.85
20–24	8.80	8.24	9.44	8.35	8.43	8.25	7.53	7.94	7.06	9.59	11.14	7.83	8.57	8.67	8.46
25–29	8.56	8.49	8.64	8.14	7.82	8.47	7.94	7.38	8.52	8.56	8.21	8.93	8.93	8.60	9.27
30–34	6.12	7.13	5.08	6.44	6.64	6.23	6.44	6.22	6.67	7.17	7.88	6.45	7.50	7.88	7.11
35–39	6.95	9.32	4.56	6.37	7.23	5.49	6.32	7.33	5.30	5.76	6.75	4.76	6.71	7.38	6.03
40–44	7.29	9.41	5.15	6.92	9.08	4.76	8.18	10.48	5.87	8.35	10.38	6.31	7.36	8.01	6.70
45–49	10.78	13.06	8.48	11.59	14.13	9.02	10.97	14.09	7.84	9.40	12.03	6.75	10.04	12.42	7.64
50–54	13.56	15.73	11.41	14.93	18.49	11.41	16.14	18.91	13.40	17.30	20.92	13.72	16.01	19.92	12.15
55–59	22.66	25.13	20.27	21.22	22.34	20.13	19.55	20.36	18.78	22.95	21.90	23.97	22.12	24.32	19.99
60–64	24.88	23.37	26.31	26.49	24.98	27.91	27.86	29.52	26.31	29.97	31.03	28.97	28.23	30.94	25.68
65–69	28.36	28.80	27.99	32.94	32.28	33.51	27.43	27.72	27.17	29.25	28.68	29.73	24.78	25.80	23.90
70–74	21.20	22.89	19.93	21.33	19.77	22.51	20.75	22.14	19.71	22.80	22.44	23.07	19.48	19.62	19.37
75–79	13.00	13.39	12.77	13.56	12.17	14.40	14.11	14.61	13.81	16.78	15.34	17.67	12.91	11.93	13.51
80–84	6.55	8.06	5.86	8.40	11.83	6.84	8.90	11.83	7.57	11.25	12.90	10.50	11.08	12.90	10.26
85–89	4.79	8.10	3.55	3.69	2.70	4.06	2.95	6.75	1.52	5.16	6.75	4.57	5.90	9.44	4.57
90–94	0.00	0.00	0.00	2.55	5.63	1.65	0.00	0.00	0.00	3.83	16.88	0.00	2.55	5.63	1.65
95–	0.00	0.00	0.00	0.00	0.00	0.00	5.84	0.00	7.01	0.00	0.00	0.00	5.84	0.00	7.01
Total	10.07	11.07	9.08	10.35	11.06	9.64	10.20	11.21	9.20	11.01	11.97	10.06	10.32	11.30	9.35

**Figure 1:**
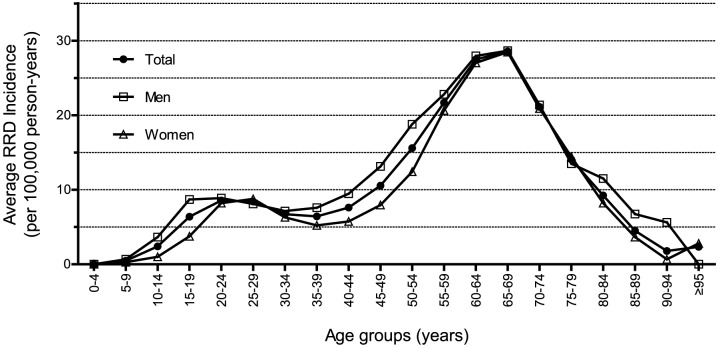
The Average Annual Incidence (per 100,000 person-years) of Rhegmatogenous Retinal Detachment (RRD) in Korean Population According to Age Groups during the Study Period from 2007 to 2011.

The pattern of the incidence of RRD across the different age groups showed bimodal distribution (Table 1, Table 2, and Figure 1). In the entire population, the highest peak RRD incidence was 28.55 per 100,000 person-years (95% CI, 27.45–29.65), which was found in the 65–69-years age group, in both men (28.66 per 100,000 person-years, 95% CI, 27.03–30.28) and in women (28.46 per 100,000 person-years, 95% CI, 26.97–29.95). The second highest peak was found in the 20–29-years age group, which was approximately 8.5 per 100,000 person-years. The male-to-female ratio was approximately 1.0 for the peak-incidence age groups, whereas the ratio increased for the other age groups.

## Discussion

In this study, we estimated the incidence of RRD by enumerating the total number of surgery-requiring RRD cases in Korea over a 5-year period. This study represents the largest nationwide population-based epidemiologic study in the world and provides highly reliable data regarding the incidence of RRD in Korea.

Our study showed relatively constant annual incidences of surgery requiring RRD during the 5-year study period and reported an average RRD incidence of 10.39 per 100,000 person-years. Although the RRD incidence of the Korean population is also comparable to the results of previous studies in other countries, the recent nationwide reports in the Netherlands and Scotland showed higher annual RRD incidences than that of our study (18.2 in 2009 and 15.28 in 2006 per 100,000 persons, respectively).[13], [15] With respect to age-specific RRD incidence, interesting differences were observed between the results of our study and these previous studies. Our results showed a bimodal distribution of RRD incidences according to age group, and the seventh decade of life showed a peak incidence of RRD, which coincides with the peak-incidence age in other countries (Table 3).[5], [8], [9], [13]–[15], [18] Although these studies had different study periods, population coverage, population size, data source, and age-group categories, an increasing trend was observed in RRD incidences with age. In all the studies, RRD incidences were similar from 0 to 49 years of age, but significant differences in RRD incidences were observed in age groups above 50 years. As stated above, both Caucasians and Asians showed a peak RRD incidence at 50–79 years of age; however, Caucasians (studies in the Netherlands,[13] Scotland,[12] Brazil,[10] New Zealand,[9] and United States[14]) had significantly higher RRD incidences at 50 years and over than did the Asians (studies in Korea, China,[7], [8] Singapore,[6] and Japan[5]). Myopia is more frequent and more severe in Asians than in Caucasians, and people with myopia develop posterior vitreous detachment (PVD) earlier and more frequently.[19]–[21] PVD typically develops at 60 years of age in Caucasians and may play a crucial role in occurrence of RRD.[22], [23] Myopia itself is one of the major risk factors of RRD.[11], [24], [25] The ethnic differences in the prevalence and degree of myopia and PVD might cause the discrepancy in the age groups showing peak incidence of RRD. The bimodal distribution of RRD incidences in Asian countries (China, 1999; Japan, 1990), including Korea, can also be attributed to myopia-induced PVD during early life.

**Table 3:** Summary of Previous Studies, by Country, reporting Rhegmatogenous Retinal Detachment Annual Incidence (per 100,000 persons).

Country	Korea	Netherlands^13^	Scotland^15,^ a	Brazil^10^	New Zealand^9^	China^8^	China^7^		Singapore^6^		Japan^5,^ b
Coverage	Nationwide	Nationwide	Nationwide	Part of nation	Part of nation	Part of nation	Part of nation		City-state		Part of nation
Period	5 years (2007–2011)	1 year (2009)	10 years (1987–2006)	1 year (2003–2004)	1 years (1997–1998)	1 year (1999)	4 years (1996–1999)		4 years (1993–1996)		1 year (1990)
Data	Claim database	Hospital records	Claim database	Hospital records	Hospital records	Hospital records	Hospital records		Claim database		Hospital records
Population^c^	49.8	16.5	5.1	1.2	1.2	6.6	0.1		2.7		1.8
Age Group	Incidence of Rhegmatogenous Retinal Detachment										
0–4	0.01	0.00	1.64	0.9	1.5	0.38				0.5	
5–9	0.48	0.10					3.8	2.3			
10–14	2.40	0.92	3.14	2.4	2.8	3.81				5.0	
15–19	6.38	1.39									
20–24	8.57	3.41	6.78	6.6	9.6	6.51				10.3	
25–29	8.43	3.73					12.6	7.8			
30–34	6.73	2.97	9.12	8.7	4.0	4.70				5.5	
35–39	6.42	5.26									
40–44	7.62	8.80	13.28	13.3	8.4	7.05				7.5	
45–49	10.56	16.52					18.6	23.4			
50–54	15.59	32.82	27.64	34.0	22.9	13.32				15.6	
55–59	21.70	52.45									
60–64	27.49	51.51	64.42	49.9	49.7	22.15				22.7	
65–69	28.55	48.95									
70–74	21.11	47.89	35.18	38.7	32.6	15.21				18.9	
75–79	14.07	37.19					17.5	21.9			
80–84	9.24	31.23				1.49					
85–89	4.50	21.43	18.25	40.6	27.8					6.4	
90–94	1.79	16.20				0					
95–	2.34	11.88									
Total	10.39	18.19	15.28	9.2	11.7	7.88	14.4	10.5		9.8	

a Mitry et al. reported the 10-year incidence in Scotland without average annual incidence. The 2006 incidence is shown in the table.

b Sasaki et al. only reported age-group incidences for phakic retinal detachments (RD). An annual RD incidence of 9.8 phakic RDs per 100,000 persons and 10.4 phakic, aphakic, or blunt trauma RD per 100,000 persons. The phakic RD incidence is shown in the table.

c Population was presented in millions.

Another explanation for the bimodal distribution of RRD incidence in Korea may be the differential change in refractive errors among the different age groups during the recent century. Korea has rapidly developed over the recent half century, and there have been several changes not only in the economy and politics but also in the body index such as height and weight. Similarly, the prevalence of myopia in Korean has increased over time. Yoon et al reported the prevalence of myopia in Korea on the basis of the database of the Korean National Health and Nutrition Examination Survey (KHNES) from 2008 to 2009.[26] The age-specific prevalence of myopia in Korea (definition of myopia: spherical equivalents of worse than −0.75 diopter) was 78.8% in the 12–18-years age group, 75.3% in the 19–29-years age group, 67.4% in the 30–39-years age group, 51.1% in the 40–49-years age group, 29.3% in the 50–59-years age group, 18.2% in the 60–69-years age group, and 28.4% in the over 70-years age group. The increase of the education in Asian countries including Korea over the recent half century might have an influence on the higher prevalence of myopia in the young age groups compared to the old age groups.[27] The results reported by Yoon et al could well explain the characteristics of our study population, because the KHNES was designed to represent the entire population of Korea. Low prevalence of myopia in the 50–79-years age group in Korea might be associated with the relatively low RRD incidence in the same age group in our study. Moreover, the level of physical activity may be higher in younger age groups such as the 20–29-years age group than in older age groups, which may cause blunt eye injuries. This may be one of the possible explanations for the bimodal distribution of RRD incidence.

The incidence of RRD in Korea may change in the near future according to the population projection in Korea (Table 1) as well as the age-specific prevalence of myopia. In 2007, the 25–49-years age group consisted of a larger population than the other age groups, including the 50–79-years age group. As the population aged 25–49 years becomes older and reaches the peak-incidence age, the incidence of RRD in Korea may increase continuously.

Studies regarding the difference in RRD incidence according to sex have reported inconsistent results. Although some studies reported that men had a lower incidence of RRD than did women, recent large-scale studies have consistently reported that RRD occurs more frequently in men than in women.[6], [9], [10], [12]–[15] Our study also confirmed that the incidence of RRD was higher in men than in women. Ocular trauma has been regarded as an attributable risk factor of RRD in men, because RRD secondary to ocular trauma may be more common in men.[6], [13] In this study, we excluded cases of penetrating/perforating ocular trauma, based on their diagnostic codes, but could not completely exclude blunt ocular trauma, which may explain the higher incidence of RRD in men. However, the risk of RRD attributable to ocular trauma is reportedly low.[1], [5], [6], [13] In addition, several studies, including those conducted in Asia, have shown no difference in the refractive error between the 2 sexes, and a study conducted in Singapore reported that the prevalence of high myopia was higher in women than in men.[28]–[30]

Interestingly, in our study, in contrast to the results observed in the general population, the bimodal incidence curve did not show any difference in the RRD incidence between the 2 sexes in 2 peak-incidence age groups (60–69-years and 20–29-years age groups). This result may imply that the basic pathophysiology of RRD is similar in men and women. Thus, the sex difference appears to apply only to a small proportion of the RRD incidents. Further studies are required regarding the differences in RRD incidence between the 2 sexes.

Our study has the following limitations. First, we may have underestimated the RRD incidence in Korea. We included the RRD cases that involved RRD surgeries of scleral buckling and vitrectomy, and excluded those RRD cases that involved other treatment options such as laser therapy, cryopexy, and pneumopexy. Because RRD surgeries of scleral buckling and vitrectomy are definitive treatment methods for vision-threatening RRD, the results reported in this study can be considered exclusive to vision-threatening RRD. Second, since we excluded cases of penetrating/perforating trauma and several vitreoretinal diseases that can complicate RRD, a small number of excluded cases may have involved true RRD in the affected eyes and/or fellow eyes, resulting in the underestimation of RRD incidence. Furthermore, in cases of 2 or more RRD surgeries in 1 patient, we included the case only at the first occurrence and discarded the following RRD surgeries. Thus, bilateral cases and recurrent cases were not included for the estimation of RRD incidence in our study. Furthermore, we could not access the hospital-based medical records for the validation of RRD occurrences and the review of clinical data. This may limit the accuracy of the data reported in this study. In addition, we could not assess the status of refractive errors and cataract operations in RRD patients, which are the major risk factors of RRD.[9], [11], [13], [14], [18], [24], [25]

In conclusion, we reported the nationwide incidence of surgery requiring RRD in Korea in the largest population ever studied over a 5-year period. RRD in Korea is characterized by a lower peak incidence than that in the Caucasian population and by the bimodal distribution of the RRD incidence according to age. Our data can be helpful in assessing the RRD-related socioeconomic burden and planning the ophthalmic health care policy. Future longitudinal studies should be conducted to evaluate the changes in the RRD incidence in Korea, according to alterations in the demographic structure and prevalence of myopia in Korea.
