# Correction: Five Year Nationwide Incidence of Rhegmatogenous Retinal Detachment Requiring Surgery in Korea

**DOI:** 10.1371/annotation/76b28079-ccd7-4f49-9cef-10498424a471

**Published:** 2013-12-03

**Authors:** Sang Jun Park, Nam-Kyong Choi, Kyu Hyung Park, Se Joon Woo

The name of the second author was spelled incorrectly. The correct name is: Nam-Kyong Choi. 

